# Anti-Inflammatory Activity of Exopolysaccharides from *Phormidium* sp. ETS05, the Most Abundant Cyanobacterium of the Therapeutic Euganean Thermal Muds, Using the Zebrafish Model

**DOI:** 10.3390/biom10040582

**Published:** 2020-04-10

**Authors:** Raffaella Margherita Zampieri, Alessandra Adessi, Fabrizio Caldara, Alessia Codato, Mattia Furlan, Chiara Rampazzo, Roberto De Philippis, Nicoletta La Rocca, Luisa Dalla Valle

**Affiliations:** 1 Department of Biology, University of Padova, Via U. Bassi 58/b, 35131 Padova, Italy; raffaellamargherita.zampieri@phd.unipd.it (R.M.Z.); alessiacodato93@gmail.com (A.C.); mattiafurlan95@gmail.com (M.F.); chiara.rampazzo.1@unipd.it (C.R.); 2 Department of Agriculture, Food, Environment and Forestry (DAGRI), University of Florence, Via Maragliano 77, 50144 Firenze, Italy; alessandra.adessi@unifi.it (A.A.); roberto.dephilippis@unifi.it (R.D.P.); 3 Pietro d’Abano Thermal Studies Center, Via Jappelli 5, Abano Terme, 35031 Padova, Italy; fabrizio.caldara@centrostuditermali.org

**Keywords:** cyanobacteria, exopolysaccharides, bioactive molecules, Euganean Thermal District, zebrafish, inflammation models

## Abstract

The Euganean Thermal District (Italy) represents the oldest and largest thermal center in Europe, and its therapeutic mud is considered a unique product whose beneficial effects have been documented since Ancient Roman times. Mud properties depend on the heat and electrolytes of the thermal water, as well as on the bioactive molecules produced by its biotic component, mainly represented by cyanobacteria. The investigation of the healing effects of compounds produced by the Euganean cyanobacteria represents an important goal for scientific validation of Euganean mud therapies and for the discovering of new health beneficial biomolecules. In this work, we evaluated the therapeutic potential of exopolysaccharides (EPS) produced by *Phormidium* sp. ETS05, the most abundant cyanobacterium of the Euganean mud. Specifically, *Phormidium* EPS resulted in exerting anti-inflammatory and pro-resolution activities in chemical and injury-induced zebrafish inflammation models as demonstrated using specific transgenic zebrafish lines and morphometric and expression analyses. Moreover, in vivo and in vitro tests showed no toxicity at all for the EPS concentrations tested. The results suggest that these EPS, with their combined anti-inflammatory and pro-resolution activities, could be one of the most important therapeutic molecules present in the Euganean mud and confirm the potential of these treatments for chronic inflammatory disease recovery.

## 1. Introduction

The therapeutic Euganean thermal mud is a unique product of the Euganean Thermal District (Italy) that represents the largest and oldest thermal center in Europe. The application of the therapeutic mud, whose beneficial effects have been documented since Ancient Roman times, is recognized by the Italian Health System as a healing treatment for arthro-rheumatic diseases. The beneficial mud is obtained by a specific maturation procedure that can be considered as an ancient biotechnological process. This process is now coded by a protocol [1] to be followed to obtain the “Mature Mud AOC” certification. The mud maturation process begins when virgin clay, obtained from the lakes of Arquà Petrarca (Padova, Italy), is laid in open air tanks or silos of the different thermal Spas, and maintained there for a period of at least two months, constantly covered by a layer of flowing thermal water, at the indicated temperature of 38–40 °C. This maturation procedure allows a microbial community, mainly represented by cyanobacteria [1,2], to grow on the mud surface. Microorganisms, embedded in a thick polysaccharidic matrix, generate a green biofilm that indicates the correct mud maturation. Before using the mud for therapies, this is mixed and put in tanks in which thermal water at nearly 60 °C is present, in order to reduce the natural microbial load and maintain the fluidity of the product.

Some studies contributed to defining the properties of this mud, suggesting that its effectiveness is not only due to the heat and the thermal water proprieties, but also to substances produced by cyanobacteria [3,4,5]. Cyanobacteria are known to synthesize a large variety of high-value bioactive compounds, including substances with UV protection, antiviral, antibacterial, and anticancer activities [6,7]. Thus, the investigation of the therapeutic properties of high-value molecules produced by the cyanobacteria growing on the Euganean thermal muds represents an important goal for scientific validation of this therapeutic treatment and for the discovery of new health beneficial biomolecules. So far, the anti-inflammatory activity of lipids such as monogalactosyldiacylglycerol (MGDG) and digalactosyldiacylglycerol (DGDG), produced by *Phormidium* sp. ETS-05, the most abundant cyanobacterium species of the mature mud biofilms, has been demonstrated [3,4,8,9,10]. For this reason, *Phormidium* sp. ETS-05 is considered the target species of the mud maturation process, and the effectiveness of its principles led to obtaining a European Patent of the mud’s therapeutic efficacy [11].

However, considering that a major component of mud biofilms is represented by the microbial polysaccharidic matrix, we deemed it of interest to investigate a possible therapeutic role of these macromolecules of biological origin. Exopolysaccharides (EPS) are polymers released by the microbial consortia of biofilms. They form an extremely hydrated (98% water) gel that participates in the establishment of microorganism aggregates, allowing their interactions, the capture of resources, and the tolerance of environmental stress. Depending on the habitat, biofilms can include prokaryotic and eukaryotic microorganisms, such as archaea, bacteria, cyanobacteria, microalgae, and fungi.

EPS released by bacteria and microalgae are already recognized for their anti-oxidant and anti-inflammatory activities [12,13,14,15,16,17], while a limited number of studies investigated these therapeutic activities in cyanobacteria [18,19,20,21,22].

Inflammation is a physiological defense mechanism, mediated by inflammatory cells, to protect the body from tissue injury and infection and to restore tissue homeostasis. In response to inflammatory stimuli, inflammatory cells release pro-inflammatory mediators (cytokines) that initiate and enhance the acute phase of the response. Generally, this stage is compensated by endogenous anti-inflammatory molecules produced to reduce the severity and extent of this process. Recruitment of neutrophils and the peak of inflammation are then followed by the resolution phase [23]. During the resolution phase, neutrophils that were recruited to the damage site need to be removed through apoptosis or by reverse migration outside the inflamed region [24]. Although the inflammatory response is protective, chronic and uncontrolled inflammation can have detrimental effects such as excessive tissue damage and can contribute to the pathogenesis of diseases, like rheumatic diseases, asthma, cancers, and cardiovascular diseases [25]. In osteoarthritis (OA), the degenerative process leads to progressive joint inflammation, destruction of the articular constituents, functional disability, and pain [26]. On the other hand, the long-term use of synthetic anti-inflammatory drugs, like analogs of cortisol, can result in adverse side effects.

Zebrafish (*Danio rerio*) is today a widely accepted vertebrate model for development, in vivo screening of bioactive molecules, and biomedical studies [27]. The major advantages of this model organism include its small size, high fecundity, transparency, availability of genetic tools (transgenics and mutants), and consistent physiological similarity to other vertebrates [28]. Currently, zebrafish represents a useful model also for studying inflammation and immune responses. Although the adaptative immune system is functional only at late larval stages [29], the innate one develops during embryogenesis to respond to outer environment exposure just after hatching [30].

Therefore, zebrafish embryos and larvae are now widely accepted for the in vivo analysis of antioxidant and anti-inflammatory molecules [31,32].

In this study, we aimed to analyze the chemical components of EPS extracted from pure cultures of *Phormidium* sp. ETS-05, their toxicity potential on cells and zebrafish development, and their activities on anti-inflammatory and pro-resolution responses to chemical- and injury-induced zebrafish inflammation models. We also evaluated the beneficial effects of *Phormidium* EPS treatment on the developmental delays produced by the inflammatory processes, and we confirmed the potential benefits of these molecules by molecular expression analysis.

## 2. Materials and Methods

### 2.1. Cultivation of Phormidium sp. ETS05 and Extraction of Exopolysaccharides

The original strain of *Phormidium* sp. ETS05 [8] was already present in our laboratory due to the collaboration with the Pietro d’Abano Thermal Studies Center. *Phormidium* sp. ETS05 was cultured in sterile liquid BG11 medium [33] in flasks for one month, starting form a freshly prepared inoculum. The temperature was kept stable at 30 ± 1 °C, and continuous light was provided at an intensity of 20 μmol photons m^−2^ s^−1^.

The supernatant was separated from the culture biomass by centrifugation (1500× *g*, 30 min, 4 °C). EPS were dialyzed using a 3.5 kDa cut-off membrane (Spectrum™ Spectra/Por™; Thermo Fisher Scientific, Waltham, MA, USA) for 72 h at 4 °C. The product obtained was therefore lyophilized in a freeze-drier, the polysaccharides quantified using the Dubois method [34], and then, dissolved in DMEM complete medium or fish water (50×: 25 g Instant Ocean, Acquarium systems, SS15-10) for in vitro and in vivo experiments.

### 2.2. EPS Characterization

EPS of the *Phormidium* sp. ETS05 culture were stained with Alcian blue (in 3% acetic acid, pH 2.5) and observed with a Leica DM5000 B photomicroscope (Leica Microsystems, Wetzlar, Germany) equipped with a Leica DCF425 C camera (Leica Microsystems).

For determining monosaccharidic composition, the extracts were hydrolyzed adding 1 mL of extract to 1 mL of 4 N trifluoroacetic acid (TFA) in screw-cap vials, for 120 min at 120 °C. Afterward, the excess of TFA was removed by drying on a rotary evaporator and resuspending in deionized water. This operation was repeated three times for each sample [35]. For sulfate analysis, the lyophilized EPS were hydrolyzed in 2 M HCl at 100 °C for 2 h, centrifuged after cooling, and the supernatant was analyzed by ion-exchange chromatography [36].

Both monosaccharides and sulfate analyses were performed using a Dionex ICS-2500 system chromatograph. For monosaccharide composition, the extracts were analyzed using a Dionex ICS-2500 ion exchange chromatograph (Dionex, Sunnyvale, CA, USA) equipped with an ED50 pulsed amperometric detector operating with a gold working electrode (Dionex) and a CarboPac PA1 column of 250-mm in length and a 4.6-mm internal diameter (Dionex). Eluents used were HPLC-grade water (A), 0.185 M sodium hydroxide (B), and 0.488 M sodium acetate (C). In the first stage of the analysis (from injection time to 20 min), the eluent consisted of 90% A and 10% B; in the second stage (from 20 to 30 min), the eluent consisted of 50% B and 50% C; in the final stage (from 30 to 60 min), the eluent was that of the first stage. The flow rate was kept at 1 mL min^−1^. Peaks for each sugar were identified based on the retention time of known standards [35]. For sulfate analysis, the system was equipped with a continuously regenerated anion-trap column (USA), a continuous anionic self-regenerating suppressor, a conductivity detector (ED50), an IonPac PA11 4 × 250 mm column (Dionex, Sunnyvale, CA, USA), and a reagent-free Dionex system producing high-purity 50 mM KOH at a flow rate of 2 mL/min. Sulfate solutions (1 to 10 mg/L; Fluka, Switzerland) were used as standards [36].

### 2.3. Zebrafish Lines

All fish were maintained and reared according to standard guidelines, as well as fish breeding for egg production, collection, and staging [37]. Embryos were raised in fish water in Petri dishes at 28 °C. Embryonic and larval stages were expressed in hours post fertilization (hpf) or days post fertilization (dpf). Adult zebrafish were not sacrificed for this study. All experiments were performed on embryos/larvae before the free-feeding stage and did not fall under animal experimentation laws according to EU Animal Protection Directive 2010/63/EU.

Zebrafish transgenic line Tg(8×Hs.NFκB:GFP,Luciferase)^hdb5^ (NFκB:GFP,Luc) was used to visualize and quantify the activation of nuclear factor NF-κB signaling in response to inflammatory stimuli [38]. The Tg(lys:DsRed)^nz50^ (lysC:DsRed) transgenic line, in which lysozyme C promoters drove the expression of red fluorescent protein in myeloid leukocytes, was used to visualize these cells [39].

### 2.4. In Vitro Cell Viability Assay

To evaluate the possible cytotoxic effect of EPS extracted from *Phormidium* sp. ETS05, cell viability was measured using the cell counting Kit-8 colorimetric assay (CCK-8) (96992, Sigma). Briefly, primary human skin fibroblasts, available in our laboratory (called WT1) [40], were seeded and allowed to adhere to 96 well plates at an initial density of 6 × 10^3^/well in DMEM medium plus 10% fetal calf serum, for 24 h. Before being distributed to cells, EPS were added directly to the medium and then filter-sterilized through a 0.2 µm filter. The cell medium was then replaced with fresh medium containing different concentrations of EPS (from 25 to 200 μg/mL), and the cells were incubated for 24 and 48 h. Experimental concentrations were chosen based on available literature on EPS [41,42,43]. Cells treated only with culture medium or with Triton X served as control groups. At the end of the incubation periods, cells were washed with PBS twice and then replaced with 100 μL of fresh DMEM medium. Finally, 10 μL of CCK-8 solution were added to each well and incubated for 1 h at 37 °C. Absorbance was measured using a microplate reader at a wavelength of 450 nm (Beckman Coulter DTX 880 Multimode Detector; Analytical Instruments). The absorbance in the control group was regarded as 100% cell viability. The percentage of viability was calculated using the formula: “[Mean OD (optical density) of treated cells—background absorbance/mean OD of untreated cells (control)—background absorbance] × 100”. All controls and samples were measured by three independent experiments performed in triplicate. The data presented represent the mean of all measurements.

### 2.5. Zebrafish Embryo/Larvae Developmental Toxicity Assay

The fish embryo acute toxicity test (FET) was performed according to the Organization for Economic Co-operation and Development (OECD, Paris, France) Guideline No. 236 (2013) [44] and used to analyze EPS.

Briefly, normally developed 6 hpf embryos were transferred into 24 well plates (1 embryo in 1 mL solution/well) and incubated with different concentrations (from 6.25 to 100 μg/mL) of EPS. The embryo medium was changed every 24 h with a new solution of EPS. For each specific concentration, twenty embryos were individually incubated with EPS, whereas the remaining 4 wells were used as internal water controls. The negative control (fish water) and positive control (1.5% Et-OH) were also tested. As EPS are soluble in water, no solvent control was necessary. The plates were then incubated in a temperature-controlled incubator at 28  ±  1 °C.

The developmental status of the zebrafish embryos and larvae was monitored daily at specified time points (24, 48, 72, and 96 hpf) under an inverted optical microscope, and four target outputs were recorded as indicators of lethality: coagulation of fertilized eggs, lack of somite development, non-detachment of the tail-bud from the yolk sac, and absence of heartbeat (OECD, 2013). Survival rates (percentages) and hatching were determined from the total numbers of living embryos remaining.

### 2.6. Co-Cultivation of Phormidium sp. ETS05 and Zebrafish to Evaluate Cyanobacterium Toxicity

Wild-type zebrafish embryos at 6 hpf and larvae at 3 dpf were distributed in Petri dishes containing *Phormidium* sp. ETS05 culture. The initial concentrations of *Phormidium* were 0.1, 0.2, and 0.3 (OD value), while fish water and BG11 medium were used as negative controls. The exposure period was 5 days for larvae treated starting from 6 hpf and 2 days for those treated from 3 dpf. Fish were observed daily in order to assess survival rates [45]. At the end of the co-culture, zebrafish morphometric analyses were also performed.

### 2.7. Chemicals Treatments and Caudal Fin Amputation of Zebrafish Larvae

The chemical and injury-induced inflammation models used are summarized in Figure S1. Drug treatments were performed using the bath immersion method. Copper sulfate pentahydrate (CuSO_4_·5H_2_O) (Merck KGaA, Darmstadt, Germany) was freshly made in bi-distilled water. Zebrafish larvae were exposed to 20 μM CuSO_4_·5H_2_O for 2 h, to allow absorption and induction of systemic inflammation. After four 15 min washes with fish water, larvae were incubated with EPS. Each treatment was performed three times with 12–15 larvae per replica.

Dextran sulfate sodium (DSS, 40,000 MW, Sigma-Aldrich, St. Louis, MO, USA, 42867) was prepared in fish water at the 0.5% (*w*/*v*) working dose starting from a 10% (*w*/*v*) stock concentration, freshly made to avoid DSS solution quality variation due to decomposition of the product. Inflammation was induced in 3 dpf larvae that were exposed to DSS by immersion for 24 h. After DSS removal, larvae were divided into two groups: one treated with EPS and the other maintained in regular fish water (control). Each treatment was performed three times with 12–15 larvae per replica.

Caudal fin amputation, posterior to the notochord, was performed on 3 dpf anesthetized larvae (tricaine, 0.1 mg/mL), with a sterile scalpel under a stereo microscope. Caudal fin amputated larvae were then divided into five groups: pre- and post-treatment with EPS, pre- and post-treatment with dexamethasone (DEX, Sigma-Aldrich, D4902), and tail-cut only. DEX was used as a positive control for the known anti-inflammatory and pro-resolution effects of this molecules. Larvae that were not exposed to the severing procedure were used as a control group. Each treatment was performed three times with 12–15 larvae per replica. DEX was dissolved in ethanol and added to samples to give a final concentration of 10 µM. Control fish were bathed in fish water containing vehicle (0.1% ethanol).

### 2.8. Analysis of Luciferase in NFκB:GFP,Luc Larvae

To analyze NF-κB activity in the different inflammatory models used, single larvae in the NFκB:GFP,Luc heterozygous background were transferred into individual wells of a 96 multiwell plate in 100 μL fish water (devoid of methylene blue) and supplemented with 0.5 mM D-luciferin potassium salt solution (GoldBio, St. Louis, MO, USA). The plate was then sealed using an adhesive sealing sheet. Bioluminescence from each larva was then assayed at 28 °C using the EnVision bioluminescence reader with enhanced luminescence sensitivity (PerkinElmer, Waltham, MA, USA).

### 2.9. Analysis of lysC:DsRed Transgenic Larvae

For imaging of neutrophils’ recruitment to the damage site, 3 dpf larvae in the lysC:DsRed heterozygous background were analyzed at 4 h post amputation (hpa) under a Leica M165 FC fluorescence stereomicroscope (Leica Microsystems). To quantify the number of neutrophils recruited to the wounded area, the cells in a defined area of the tail (see Figure S2) were counted manually.

### 2.10. Zebrafish Operculum Area Analysis

Operculum area was determined in 5 dpf zebrafish larvae after treatment with CuSO_4_·5H_2_O followed by EPS recovery of inflammation, following the indication of Tarasco and co-worker (2017) [46]. Briefly, five days post fertilization larvae were euthanized, stained for 15 min at room temperature with 0.01% alizarin red S (AR-S, A5533, Sigma), and then, washed twice with distilled water for 5 min. AR-S binds to calcium phosphate crystals and fluoresces in the red channel, allowing for a rapid valuation of zebrafish ossified elements.

Larvae were placed in a lateral plane on 2% agarose gel, and AR-S fluorescence was imaged under a fluorescence stereomicroscope (Leica Microsystems) equipped with a green filter. The mineralized area of the operculum was determined from the morphometric analysis of the fluorescence images using ImageJ software and normalized with the area of the head following the indication of [46].

### 2.11. Morphological Analysis and Image Processing

For imaging, embryos and larvae were anesthetized and positioned laterally to acquire images of the eye region and the whole body. Endpoints measured were eye area, total larval length (snout to tail), and swim bladder perimeter area. All parameters were processed with the ImageJ software and compared to those of the controls. All measurements were done in digital micrographs taken with a Leica M165 FC stereoscopic microscope (Leica Microsystems) equipped with a Leica DCF7000 T digital camera (Leica Microsystems).

### 2.12. RNA Isolation, cDNA Synthesis, and Expression Analyses

For expression analysis, total RNA was extracted from pools of 15–20 larvae with TRIzol reagent (Thermo Fisher Scientific, 15596026). Poly(A) mRNA was purified from 5 μg of total RNA with Dynabeads “mRNA direct kit” (Thermo Fisher Scientific, 61011) and used for cDNA synthesis with SuperScript™ IV Reverse Transcriptase (Thermo Fisher Scientific, 18090010) according to the manufacturer’s protocol. PCRs were performed with the SYBR green (Bio-Rad Laboratories, *Hercules, CA, USA*) method in a CFX96 Touch Real-Time PCR Detection System (Bio-Rad Laboratories). Data were normalized to the expression of β*-actin* to standardize the results by eliminating variation in mRNA and cDNA quantity and quality. The annealing temperature for PCR ranged from 58 to 60 °C, depending on the primer set used. The cycling parameters were 95 °C for 10 min, followed by 45 cycles at 95 °C for 30 s, and annealing-extension for 30 s. No amplification products were observed in negative controls and no primer-dimer formations in the control templates. qPCR results were analyzed using the ΔΔCt method using the Bio-Rad CFX Manager software Version 3.1 (Bio-Rad Laboratories). Three biological replicas of the experiments were performed, and all reactions were done as technical triplicates. Primer sequences are reported in Table S1.

### 2.13. Statistical Analyses

Statistical analysis was performed using Graph Pad Prism V7.0 software (GraphPad software). Data are presented as the means ± SEM or means ± SD (indicated for each figure). The statistical analysis of comparison between control and treated samples was performed with one-way ANOVA followed by Tukey’s multiple comparison test, except for some experiments in which unpaired *t*-tests with Welch’s correction were used, as indicated in the figure legends. The *p*-values were shortened with the following symbols: * *p* < 0.05, ** *p* < 0.01, *** *p* < 0.001, **** *p* < 0.0001 (significance was set at *p* < 0.05). Experiments were repeated at least three times, except where differently specified in the text or legends.

## 3. Results

### 3.1. Chemical Characterization of the EPS Produced by Phormidium sp. ETS05 Pure Culture

**Figure 1:**
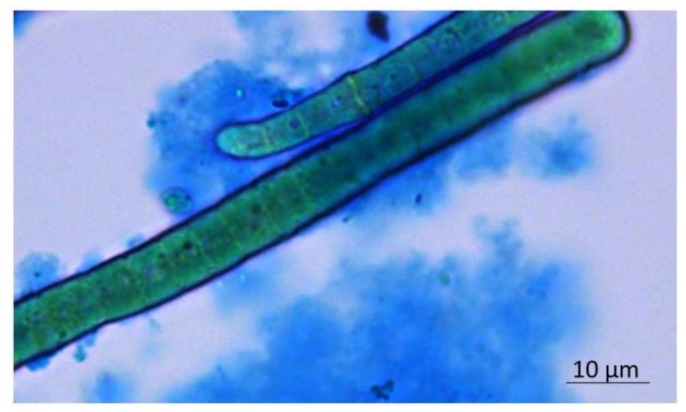
Visualization of *Phormidium* EPS through the bright-field image of Alcian-blue-stained culture.

The EPS produced by a pure culture of *Phormidium* sp. ETS05 were analyzed in terms of monosaccharides composing the polymer and the amount of sulfate groups present (Table 1). The EPS resulted in being composed of 10 monosaccharides, in particular: deoxy-sugars, fucose and rhamnose; pentoses, arabinose and xylose; aldo-hexoses, glucose, galactose, and mannose; amino-sugars, glucosamine; and acidic sugars, galacturonic and glucuronic acids. The three main components were xylose, rhamnose, and glucose. The polymer showed the presence of negative charges (as shown by Alcian blue stain, in Figure 1), confirmed by the presence of acidic sugar residues and a high percentage of sulfate groups.

### 3.2. Phormidium EPS Treatment Does Not Interfere with In Vitro Cell Viability of Human Skin Fibroblast Cells

**Figure 2:**
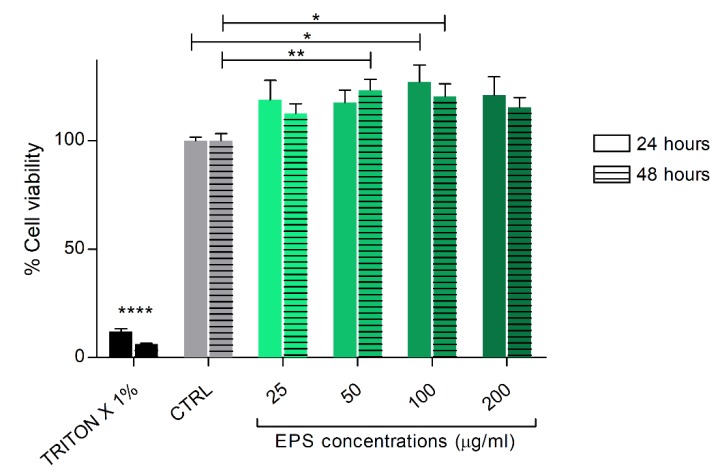
Cell viability analysis after HSF cells treatment with EPS from *Phormidium* sp. ETS05. The cells were cultured with the indicated concentrations of EPS for 24 and 48 h. Cell viability was assessed. Data are shown as the mean ± SEM of three independent experiments, each carried out in triplicate. Statistical analysis was performed using GraphPad Prism 7 (one-way ANOVA, Tukey’s multiple comparison test). Statistical significance: * *p* ≤ 0.05, ** *p* ≤ 0.01, **** *p* ≤ 0.0001

To exclude cellular toxicity, human skin fibroblasts (HSF) were treated with different concentrations of *Phormidium* EPS (25–200 μg/mL) for 24 and 48 h, and cell viability was evaluated using the CCK-8 assay. EPS did not show any cytotoxicity toward HSF cells with all the concentrations tested. On the contrary, the analysis showed an increase of HSF cells’ viability with EPS treatments from 25 to 100 μg/mL. The increase was statistically significant with the 50 and 100 μg/mL doses (Figure 2).

### 3.3. Phormidium EPS Treatment Has No Toxicological or Teratogenic Effects on Zebrafish Development

In vivo toxicological assessment of *Phormidium* EPS was conducted before the evaluation of its anti-inflammatory and pro-resolution potential, following the indication of the FET test (2013). The treatments with different concentrations of EPS (6.25–100 μg/mL) started at the 6 hpf stage and ended at 96 hpf. The mortality of the negative and positive control at 96 hpf was 0% and 40%, respectively, thus demonstrating the achievement of the FET test acceptance criteria (OEDC 236). The survival and hatching rates were not affected by exposure to any EPS concentration used, indicating that, even at the higher concentrations, EPS extract did not disturb zebrafish development. Moreover, the lack of negative changes related to the morphological outputs required by the FET test (coagulation of fertilized eggs, lack of somite development, non-detachment of the tail-bud from the yolk sac, and absence of heartbeat) suggested that no toxicity or teratogenic effects could be linked to the compounds tested.

**Figure 3:**
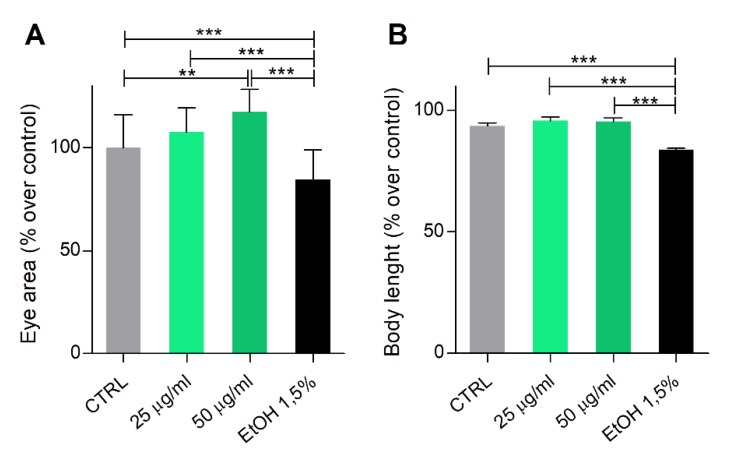
*Phormidium* EPS treatment has no toxic effects during zebrafish development. Zebrafish embryos were treated with EPS from 6 to 96 hpf, following the indication of the fish embryo acute toxicity test (FET) test. Graphs report results obtained with 25 and 50 µg/mL concentrations, relative to the positive effects on: (**A**) the eye’s area at 1 dpf and (**B**) total body length at 4 dpf. Data are shown as the mean ± SD. Statistical analysis was performed using GraphPad Prism 7 (one-way ANOVA followed by Tukey’s multiple comparison test). Statistical significance: ** *p* ≤ 0.01, *** *p* < 0.001.

This result prompted us to focus our analyses on the developmental positive effects of *Phormidium* EPS treatment. Therefore, digital micrographs of embryos and larvae were analyzed with the ImageJ software to measure the eye’s area at 1 dpf and total body length at 4 dpf. The eye area was found significantly greater with EPS at the 50 μg/mL concentration, while body length showed a positive trend, although not statistically significant, at both concentrations (Figure 3). As expected, Et-OH treatment resulted in a significant reduction of both parameters in the surviving larvae.

Based on these results and literature data on the use of EPS extracted from other species [47,48], *Phormidium* EPS were used at a 50 μg/mL concentration.

### 3.4. Co-Cultivation of Phormidium sp. ETS05 and Zebrafish to Evaluate Cyanobacterium Toxicity

To study the possible release of toxic compounds by *Phormidium* sp. ETS05, zebrafish embryos at 6 hpf and larvae at 3 dpf were cultured together with the cyanobacteria at three starting concentrations (0.1, 0.2 and 0.3 OD value) up to 5 days post fertilization. Negative controls, based on both zebrafish in their standard medium, fish water, and zebrafish in BG11 medium, showed no difference between them. At the end of the exposure, larval survival rate was 100% for all *Phormidium* sp. ETS05 concentrations and both zebrafish stages tested. Moreover, we analyzed three morphological parameters of the larvae in order to assess their correct development. Eye area, body length and swim bladder area showed no significant difference between negative controls and larvae exposed to *Phormidium* sp. ETS05 at each concentration (Figure S3). These results confirm the lack of toxicity due to the presence of *Phormidium* sp. ETS05 and of molecules eventually released by the cyanobacterium in the medium.

### 3.5. Zebrafish Developmental Delay due to CuSO_4_·5H_2_O Treatment is Rescued by Phormidium EPS Treatment

**Figure 4:**
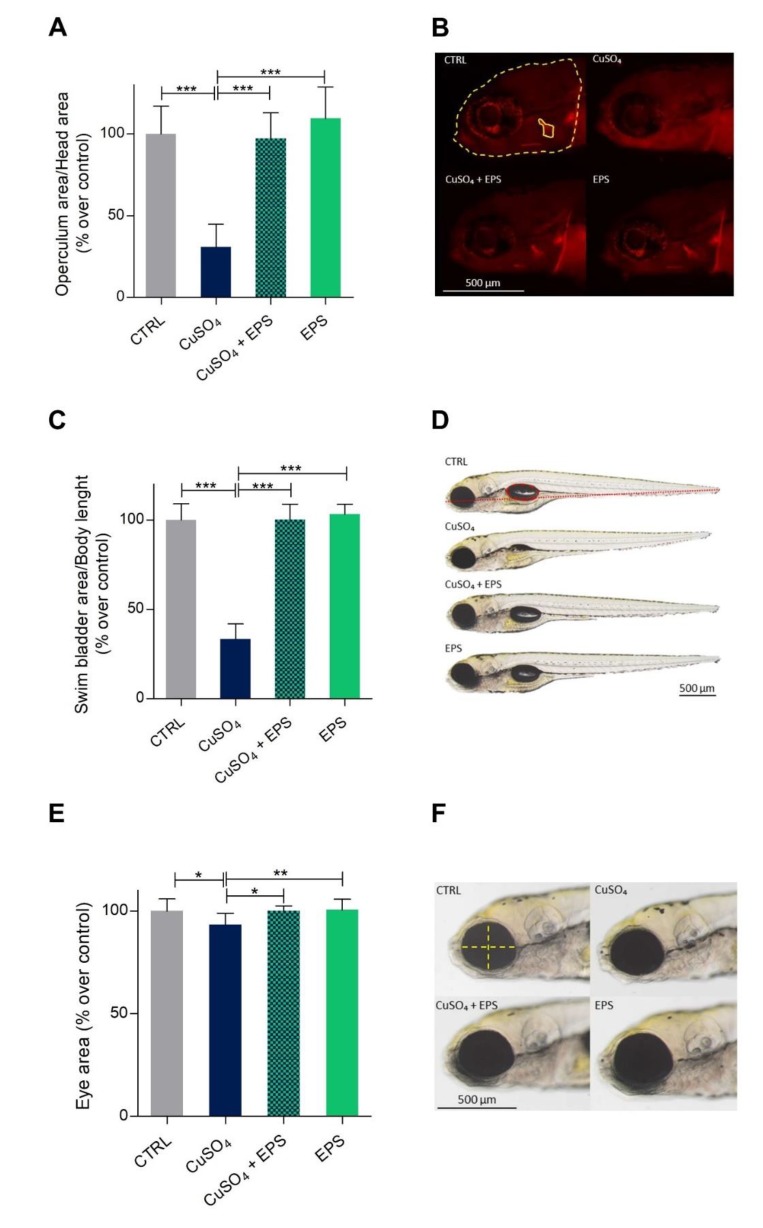
Recovering of normal developmental parameters after copper induced inflammation with *Phormidium* EPS treatment. (**A**) Reduced operculum mineralization due to copper was recovered after treatment with EPS. Operculum area was determined by normalization with the area of the head according to Tarasco and co-worker (2017) [46]. (**B**) Picture of alizarin red S (AR-S) stained 5 dpf zebrafish larvae’s head showing the area of the head and the operculum evaluated through morphometric analysis. (**C**) CuSO_4_·5H_2_O treated larvae showed consistently a deflated swim bladder at 5 dpf, which was partially recovered after treatment with EPS. (**D**) Micrographs showing reduced inflation of swim bladder induced by CuSO_4_·5H_2_O exposure and rescue due to EPS treatment. Wild-type larvae of the same ages are shown for comparison. In the zebrafish control image, the swim bladder is outlined by a red dashed line, whereas the body length by a dotted line. Scale bar = 500 μm. (**E**) CuSO_4_·5H_2_O treated larvae showed significant reduction of eye dimension at 5 dpf, which was recovered after treatment with EPS. (**F**) Micrographs showing reduced eye dimension after CuSO_4_·5H_2_O exposure and rescue due to EPS treatment. Wild-type larvae of the same ages are shown for comparison. Scale bar = 500 μm. The data represent the mean ± SD of three independent experiments conducted with 12–15 larvae. Statistical analysis was performed using GraphPad Prism 7 (one-way ANOVA followed by Tukey’s multiple comparison test). Statistical significance: * *p* < 0.05, ** *p* < 0.01, *** *p* ≤ 0.001.

In this study, copper treatment was used to induce a general inflammatory response [49,50] and to determine the protective effects of *Phormidium* EPS on the toxicity of this heavy metal. To avoid the chelating effect of EPS of bipositive cations [51], the larvae, after being treated with copper, were washed before the application of EPS. We first analyzed the operculum area, which is among the first bony structures to ossify starting from 3 dpf. Morphometric analysis of this bone, stained with AL-S, showed that copper treatment impaired bone mineralization and slowed bone development, a result that could be used as a positive indication of the effectiveness of CuSO_4_·5H_2_O. However, treatment with *Phormidium* EPS significantly recovered the size of operculum area in CuSO_4_·5H_2_O treated larvae, as shown in Figure 4A,B.

Furthermore, since exposure to copper is known to delay swim bladder development [52], we assessed the swim bladder inflation after EPS treatment. As shown in Figure 4C,D, CuSO_4_·5H_2_O treated larvae showed partially inflated swim bladders resulting in a significant reduction of their size. Again, this developmental delay was significantly rescued with EPS. Finally, copper treatment also resulted in a significant reduction in the eye area. Once more, *Phormidium* EPS significantly reversed the negative effects of copper on this parameter (Figure 4E,F).

### 3.6. Copper Induced Inflammatory Status Can Be Recovered after Phormidium EPS Treatment

In this study, the NFκB:GFP,Luc zebrafish transgenic line was used to assess the anti-inflammatory effects of *Phormidium* EPS treatment on a copper induced inflammation model. This line allows a global quantitative evaluation of NF-κB transcription factors’ activity by bioluminescence measurement of luciferase activity, and this can be used as a readout of inflammation [38]. To test the time course of the inflammatory response that can be obtained with this transgenic line, we analyzed larvae after 9, 24, and 48 hpi (hour post injury). As shown in Figure S4, luciferase activity was only slightly increased 9 h after CuSO_4_·5H_2_O treatment, while it showed a consistent upregulation 24 and 48 hpi, in agreement with Kuri and colleagues (2017) [38], who reported a significant bioluminescent signal at 40 h post injury.

**Figure 5:**
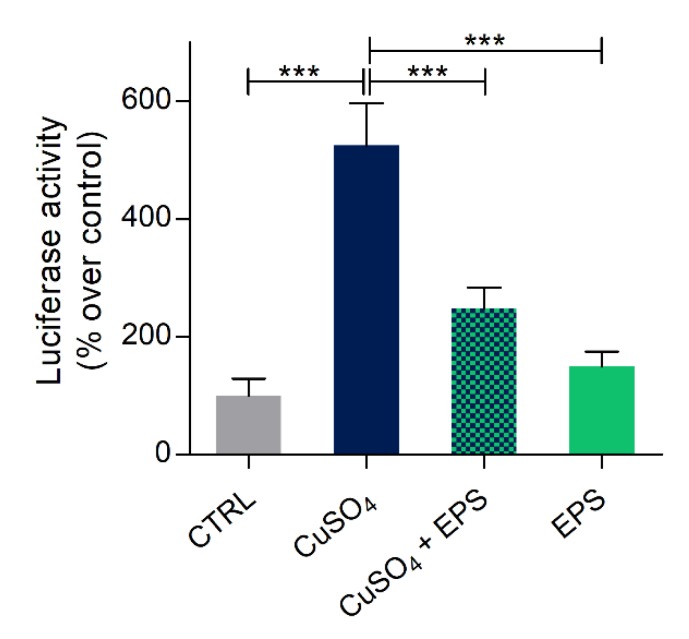
Anti-inflammatory effects of *Phormidium* EPS after copper induced inflammation in zebrafish larvae. Recovery from inflammation was analyzed with the zebrafish transgenic line NFκB:GFP,Luc. The data represent the mean ± SEM of three independent experiments conducted with 12–15 larvae. Statistical analysis was performed using GraphPad Prism 7 (one-way ANOVA followed by Tukey’s multiple comparison test). Statistical significance: *** *p* ≤ 0.001.

Based on these results, the effects of *Phormidium* EPS treatments were then analyzed in 5 dpf larvae, 48 h after inflammation induction and EPS treatment. As shown in Figure 5, luciferase activity was significantly reduced with EPS treatment. This result demonstrated that EPS treatment markedly attenuated the increase of NF-κB transcription factors’ activity linked to inflammation.

### 3.7. Phormidium EPS Can Reduce the Inflammatory Response Induced by DSS and Caudal Fin Amputation

To further validate the anti-inflammatory effects of *Phormidium* EPS, two other inflammation models were used.

**Figure 6:**
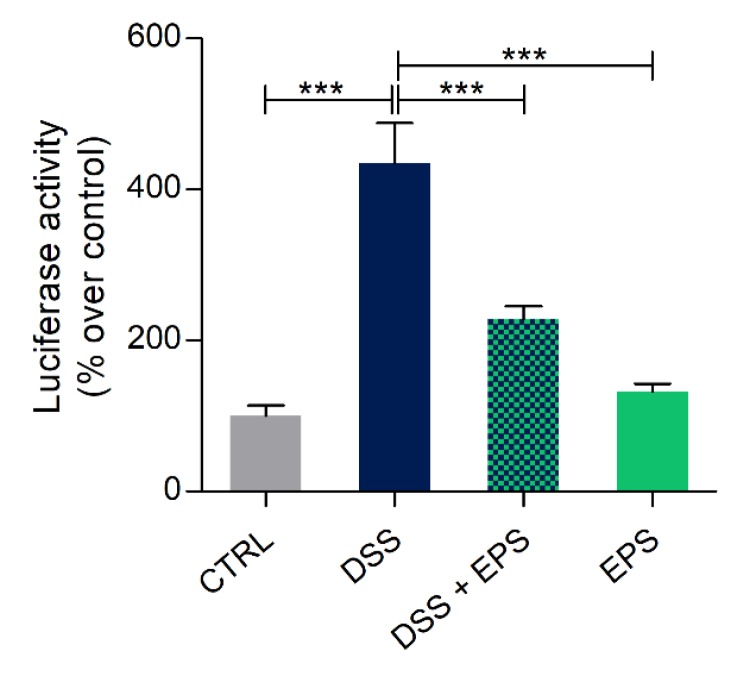
Anti-inflammatory effects of *Phormidium* EPS after dextran sulfate sodium (DSS) induced inflammation in zebrafish larvae. Recovery from inflammation was analyzed with the zebrafish transgenic line NFκB:GFP,Luc. The data represent the mean ± SEM of three independent experiments conducted with 12–15 larvae. Statistical analysis was performed using GraphPad Prism 7 (one-way ANOVA followed by Tukey’s multiple comparison test). Statistical significance: * *p* ≤ 0.05, *** *p* ≤ 0.001.

As the first alternative to copper, inflammation was chemically induced with DSS, a molecule normally used to develop an in vivo model of inflammatory bowel disease [53]. The 5-dpf DSS treated larvae in the NFκB:GFP,Luc transgenic background showed a statistically significant higher luciferase activity than control larvae. However, this increase was significantly reduced after 24 h EPS treatment (Figure 6).

Besides, local inflammation can be induced and analyzed by the amputation of zebrafish larvae caudal fin. NF-κB transcription factor activity in the NFκB:GFP,Luc transgenic larvae was analyzed 6, 24, and 48 h after tail cutting to assess the best sampling time to analyze inflammation and potential beneficial *Phormidium* EPS effects. A statistically significant increase in luciferase activity was obtained after 48 h (Figure S5), a time lapse that was then used for the subsequent analysis.

**Figure 7:**
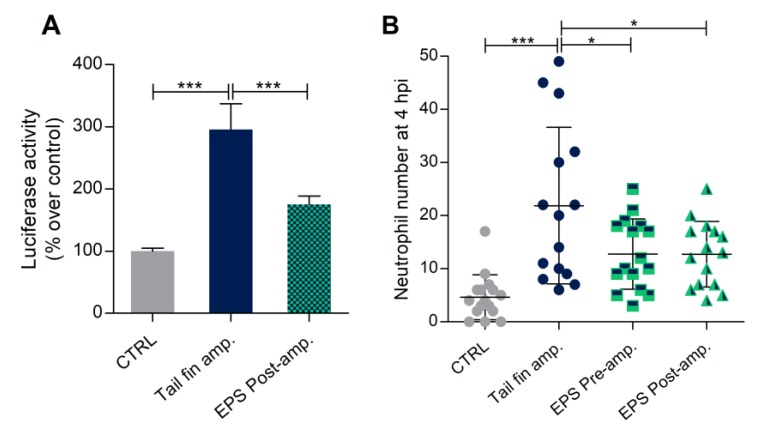
Anti-inflammatory effects of *Phormidium* EPS after caudal fin amputation induced inflammation in zebrafish larvae. (**A**) Recovery from inflammation was analyzed with the zebrafish transgenic line NFκB:GFP,Luc. EPS treatment determined a significant reduction of Luciferase activity recorded 48 h after fin amputation. (**B**) EPS treatment significantly decreased the number of neutrophils in the damaged area of 3 dpf tail fin amputated larvae in LysC:DsRed background. The data represent the mean ± SEM (**A**) and the mean ± SD (**B**) of three independent experiments conducted with 12–15 larvae. amp. = amputation. Statistical analysis was performed using GraphPad Prism 7 (one-way ANOVA followed by Tukey’s multiple comparison test). Statistical significance: *** *p* ≤ 0.001, * *p* ≤ 0.05.

As illustrated in Figure 7A, the luciferase activity in larvae exposed to EPS was significantly reduced by this treatment, confirming the anti-inflammatory potential of *Phormidium* EPS also in this model.

Moreover, the same inflammatory model was adopted with the lysC:DsRed transgenic line, in which the number of neutrophils in the wound region could be used to study the anti-inflammatory effect of *Phormidium* EPS. The analysis was performed at 3 h post amputation (hpa) (Figure S2), with both pre- and post-treatment with EPS. We found that both pre- and post-EPS treatments resulted in a significant reduction of the number of neutrophils in the inflammatory site with respect to the control group (only cut larvae) (Figure 7B).

### 3.8. Effects of Phormidium EPS on the Expression of Inflammatory Markers

**Figure 8:**
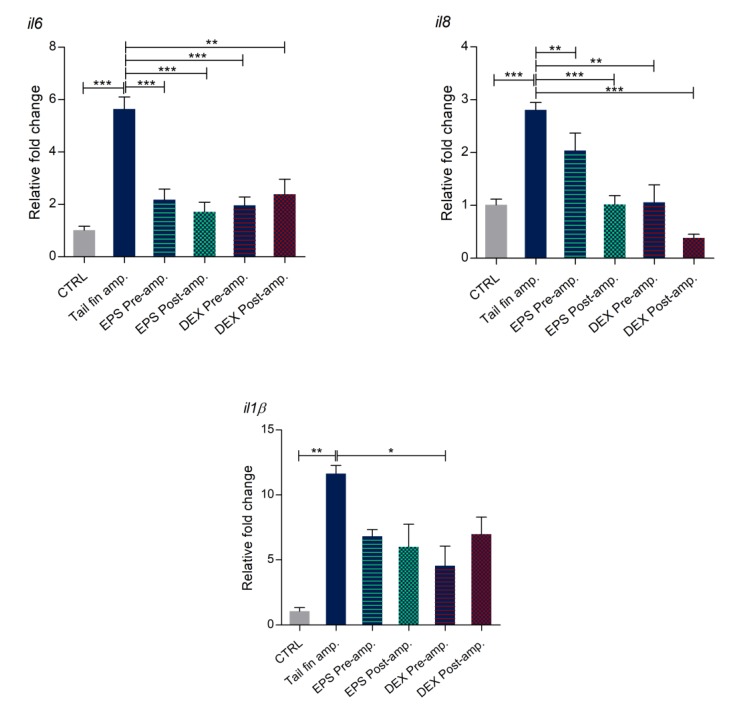

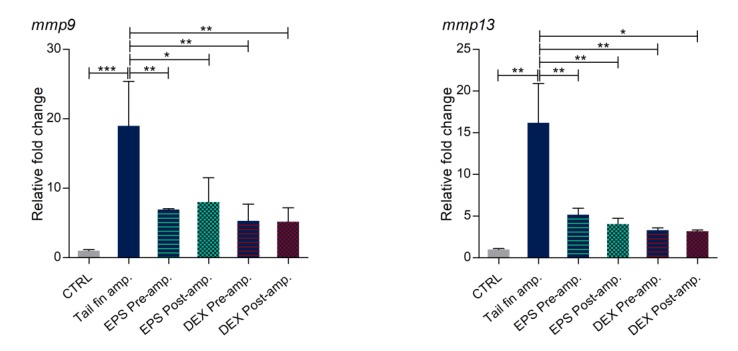
Expression of immune responsive genes in tail fin amputated larvae. qRT-PCR analysis of *il8*, *il6*, *il1b*, *mmp9*, and *mmp13* expression levels was performed with 3 dpf zebrafish larvae under basal conditions and tail cut amputation with or without dexamethasone (DEX) and EPS treatments. The data represent the mean ± SD of three independent experiments conducted with 12–15 larvae. amp. = amputation. Statistical analysis was performed using GraphPad Prism 7 (one-way ANOVA followed by Tukey’s multiple-comparison test). * *p* ≤ 0.05, ** *p* ≤ 0.01, *** *p* ≤ 0.001.

To confirm the anti-inflammatory and pro-resolution effect of *Phormidium* EPS, we analyzed the expression levels of several immune responsive genes, including pro-inflammatory cytokines (*il1b* and *il6*), chemokine (*il8*), and genes coding for matrix metalloproteinase proteins (*mmp9* and *mmp13*), in response to the tail fin amputations as the inflammatory stimulus. Our RT-qPCR results demonstrated that, in tail amputated larvae, transcription levels of all the analyzed genes were significantly upregulated when compared to control uncut larvae. In contrast, after pre- and post-DEX treatments, the expression levels were statistically reduced in all cases except for *il1b* expression in larvae treated with DEX after amputation (Figure 8). A similar response was obtained with pre- and post-EPS treatments. In this case, downregulation of *il8* (pre-treatment) and *il1b* (both pre- and post-treatment) was not significant, although the trend was the same between DEX and EPS treated larvae, thus confirming that *Phormidium* EPS exert positive effects with respect to the inflammation process by downregulation of inflammatory marker genes.

## 4. Discussion

*Phormidium* sp. ETS05 is the most abundant cyanobacterium present on the surface of Euganean mature muds. So far, its lipidic compounds MDGD and DGDG are the only recognized as bioactive molecules contributing to the therapeutic effects of mud treatments. However, considering the abundant microbial polysaccharidic matrix of mud biofilms and the growing interest in exopolysaccharides as anti-inflammatory biomolecules, we deemed it important to evaluate the anti-inflammatory and pro-resolution potential of *Phormidium* EPS in vivo.

The chemical analysis of the EPS extracted from the pure culture of *Phormidium* sp. ETS05 showed that the main sugar residues composing the polymer were xylose, rhamnose, and glucose, the sum of the three giving 64% of the total composition, while galactose, fucose, mannose, arabinose, glucosamine, and two uronic acids were present in minor amounts. A total of 10 detectable monosaccharides were identified here, which is comparable to species of the genus *Phormidium* such as the eight-to-twelve monosaccharides in *P. tenue* [54,55] or the ten in *P. versicolor* [22]. Cyanobacterial polysaccharides showing anti-inflammatory activity on various animal models, as the capsular polysaccharides from *Mastigocladus laminosus* [56,57] or sacran from *Aphanothece sacrum* [20,58], are composed of various proportions of eight main sugars, namely glucose, galactose, mannose, xylose, rhamnose, fucose, and galacturonic and glucuronic acids. These are the same core sugars that we found for *Phormidium* sp. ETS05, and we also found the additional presence of arabinose and glucosamine even if in low percentages.

The *Phormidium* sp. ETS05 EPS resulted in being highly sulfated (≈13% *w*/*w*), being among the highest values found for cyanobacterial polymers [36,59]. Similar values of sulfate content were previously detected in polysaccharides produced by cyanobacteria isolated from Polynesian mats [60], in *Nostoc carneum* [61], or in cyanoflan, a polymer isolated from marine *Cyanothece* sp. cultures [62]. High amounts of sulfate were previously reported for various cyanobacterial EPS with proven anti-inflammatory activity [63].

Overall, the chemical characteristics of *Phormidium* sp. ETS05 EPS showed the amphiphilic nature of these polymers. Indeed, on one side, deoxyhexose (rhamnose and fucose) residues introduce a certain hydrophobic/lipophilic character to the polymer, presumably contributing to the polysaccharide emulsifying properties; on the other side, the presence of sulfate groups, together with uronic acids, strongly contributes to the overall anionic character of these polysaccharides.

These characteristics may indeed be part of the success of EPS interaction with the epidermal layer.

Before investigating a possible anti-inflammatory effect, human cells and zebrafish embryos and larvae were exposed to different concentrations of *Phormidium* EPS in order to exclude any toxic effects. No reduction in survival rate or morphological abnormalities were observed, demonstrating that these molecules, even at the higher exposure concentrations used, did not have any harmful effects, not only on human cells, but also on zebrafish development. This was in agreement with other publications that demonstrated the absence of toxicity of EPS on human dermal fibroblasts [64] and on zebrafish embryos [47]. Furthermore, the exposure of zebrafish embryos and larvae to the *Phormidium* sp. ETS05 culture for five days did not lead to an increase in mortality and did not interfere with the normal development of the zebrafish.

To test *Phormidium* EPS immunomodulatory activity and to evaluate its potential in recovery from inflammation, three different in vivo zebrafish models were used in which this condition was induced chemically by CuSO_4_·5H_2_O or DSS exposures and mechanically by caudal fin amputation.

Exposure to copper can cause injuries to superficial tissues, in particular to the hair cells of the zebrafish lateral line, followed by an acute inflammatory response [49,50]. Thus, treatment of zebrafish embryos/larvae with copper is today commonly used to analyze inflammatory response [65,66]. Since cyanobacterial EPS can bind dissolved heavy metal bipositive cations such as Cu^2+^ or Pb^2+^ [51], *Phormidium* EPS was applied to copper-treated larvae only after multiple washes. The simultaneous presence in solution of EPS and copper ions could have decreased the toxicity of the heavy metal on the larvae; removing copper from the solution allowed evaluating the EPS activity without interfering factors. In agreement with already reported data [67], the efficacy of copper treatment to induce detrimental effects was demonstrated by a general delay of zebrafish development, here reported as a reduction of the swim bladder insufflation and delay in the operculum ossification. *Phormidium* EPS clearly exerted a protective effect as both parameters were statistically recovered after treatment with this molecule.

Moreover, *Phormidium* EPS exposure showed significant anti-inflammatory effects in copper through downregulation of NF-κB signaling, as demonstrated by the use of the zebrafish transgenic line NFκB:GFP,Luc [38]. After its activation by different inflammatory stimuli, the transcription factor NF-κB could regulate this process by activation of the transcription of various immunomodulator genes, like cytokines, chemokines, and invasion molecules [68].

However, deregulation of the NF-κB signaling pathway can play a fundamental role in the pathogenesis of most chronic inflammatory diseases including rheumatoid arthritis [69], cardiovascular disease, inflammatory bowel disease, and neurodegenerative diseases [68].

A reduction of NF-κB signaling was obtained also with the other two inflammatory models used in this work to further support the beneficial capabilities of *Phormidium* EPS. Exposure to DSS is normally used to recapitulate the principal features of inflammatory bowel disease, not only in mammals, but also in zebrafish [31], whereas the tail fin amputation model allows analyzing the expression of many pro-inflammatory molecules and, when associated with specific transgenic reporters, the recruitment of neutrophils and macrophages towards the damaged area [39,70].

In both inflammatory models, the NF-κB activation was significantly reduced to levels comparable to those of the controls, thus confirming the potential of *Phormidium* EPS treatment to recover from inflammation. Although more in depth analyses are required to fully understand the *Phormidium* EPS mechanisms underlying its role on inflammation, the results obtained with the three zebrafish models and the transgenic line NFκB:GFP,Luc suggested that these EPS molecules could exert their anti-inflammatory function through regulation of NF-κB signaling pathways.

Attenuation of inflammation was further verified by the analysis of neutrophil recruitment to the site of damage in the tail fin amputation inflammation model using the zebrafish transgenic line LysC:DsRed. This experimental approach allowed us to analyze the anti-inflammatory and pro-resolution effects of *Phormidium* EPS as larvae were subjected to both a pre- and a post-treatment with EPS.

The consistent reduced neutrophil number in the damaged site obtained with both treatments suggested that *Phormidium* EPS could not only act as an anti-inflammatory agent by preventing or reducing their recruitment, but also stimulate the resolution of inflammation by accelerating the clearance of these cells in order to recuperate tissue homeostasis.

Furthermore, we also demonstrated that *Phormidium* EPS showed in vivo anti-inflammatory and pro-resolution activity via downregulation of the expression of some inflammatory markers in comparison with the effects due to the administration of DEX, one of the synthetic glucocorticoids normally used in chronic inflammatory therapy [71]. Although very effective, these molecules also have many side effects that limit or advise against their prolonged use [72]. Expression analyses showed that both DEX and EPS downregulated with comparable effectiveness the amputation induced transcriptional increases of inflammatory markers such as pro-inflammatory mediators *il6*, *il1β*, and *il8* and of matrix metalloproteinases *mmp9* and *13*, which by degradation of the extracellular matrix, could facilitate the advance of cell migration and invasion [73].

Interestingly, the inflammatory markers analyzed in this work, and in particular *il6, il8*, and *mmp13*, were found to be upregulated also in osteoarthritis (OA) and rheumatoid arthritis (RA), the two most common types of arthritis [74,75,76,77,78], providing a clue to the possible mechanisms used by these molecules against these painful pathologies.

Moreover, our results indicated that *Phormidium* EPS modulate the anti-inflammation pathway through key mediators also regulated by *Phormidium* MGDG [4], suggesting that these two compounds could have a synergic healing effect in mud therapy.

Although the pathways and molecular interactions activated by EPS for the moment were not identified, downregulation of NF-κB signaling, cytokines, and inflammatory markers, the reduction of neutrophils at the damage site, and recovery of detrimental effects all provided clear evidence that EPS were effective as anti-inflammatory and pro-resolution compounds and confirmed the potential of mud treatments for chronic inflammatory disease recovery.

## Figures and Tables

**Table 1:** Monosaccharidic composition (as molar %, listed from the most to the least abundant) and sulfate groups (measured as % in weight) quantification of EPS from *Phormidium* sp. ETS05. Mean and standard deviation (st. dev.) for triplicates are reported.

Molar %	Mean	st. dev.
Xylose	28.2	1.5
Rhamnose	18.4	3.2
Glucose	18.0	0.7
Galactose	08.7	0.6
Arabinose	04.7	0.1
Fucose	07.2	0.4
Glucosamine	03.6	0.4
Mannose	06.1	0.7
Glucuronic acid	02.4	0.6
Galacturonic acid	01.4	1.5
Galactosamine	Traces	-
Fructose	Traces	-
Ribose	Traces	-
**% (w/w)**	**mean**	**st. dev**
Sulfates	13.2	2.0

## Supplementary Materials

The following are available online at https://www.mdpi.com/2218-273X/10/4/582/s1, Figure S1: Chemically and injury-induced inflammation models used, Figure S2: Caudal tail amputation site and neutrophils visualization through microscopy, Figure S3: Morphological parameters of zebrafish larvae after co-culture with *Phormidium*, Figure S4: NF-κB activation after copper treatment analyzed at three time points, Figure S5: NF-κB activation after caudal tail amputation analyzed at three time points. Table S1: Primers for Real-Time PCR.
