# Supplementary material for: Anti-Inflammatory Activity of Exopolysaccharides from Phormidium sp. ETS05, the Most Abundant Cyanobacterium of the Therapeutic Euganean Thermal Muds, Using the Zebrafish Model
Source: Biomolecules. 2020 Apr 10;10(4):582. doi: 10.3390/biom10040582 (PMC7226003; doi:10.3390/biom10040582)
Supplement: Supplementary file 1 [file biomolecules-10-00582-s001.pdf]

biomolecules

MDPI

Supplementary materials

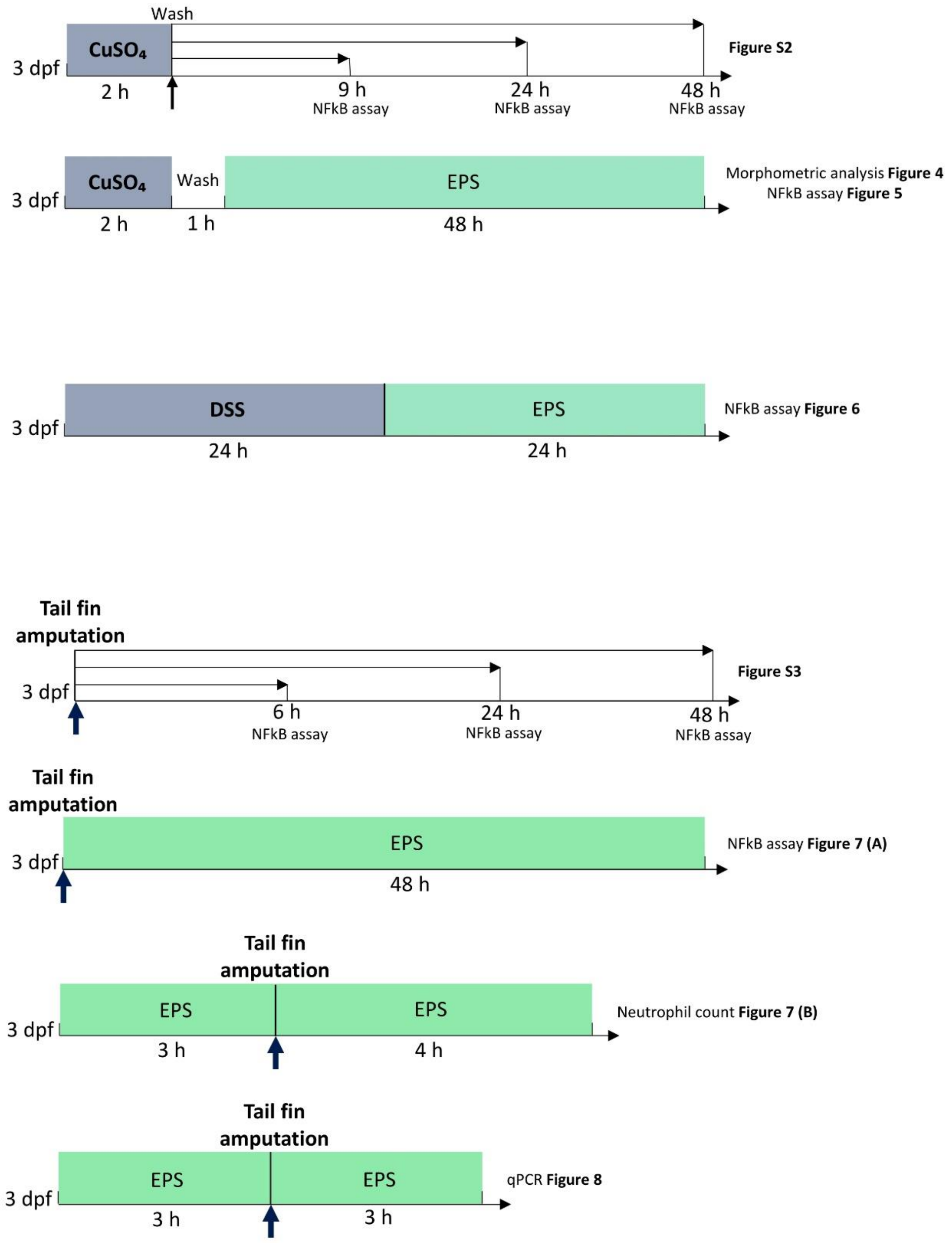

Figure S1. Schematic representation of chemically and injury-induced inflammation models used.

Table S1. Primers for Real-Time PCR

Gene	Forward (5′-3′)	Reverse (5′-3′)	Genbank acc. no.
il1b	GACATGCTCATGGCGAACG	GCAAATCGTGCATTGCAAGACG	NM_212844
il6	GTGAAGACACTCAGAGACG	GTTAGACATCTTTCCGTGCTG	NM_001261449
il8	TTGAAGGAATGAGCTTGAGAGG	TCATGGAGCAGAGGGGTC	XM_009306855
mmp9	CATTAAAGATGCCCTGATGTATCCC	AGTGGTGGTCCGTGGTTGAG	NM_213123
mmp13	ATGGTGCAAGGCTATCCCAAGAGT	GCCTGTTGTTGGAGCCAAACTCAA	NM_001290479
β-actin	TGGGTATGGAATCTTGCGGT	GTGGGGCAATGATCTTGATCT	NM_181601

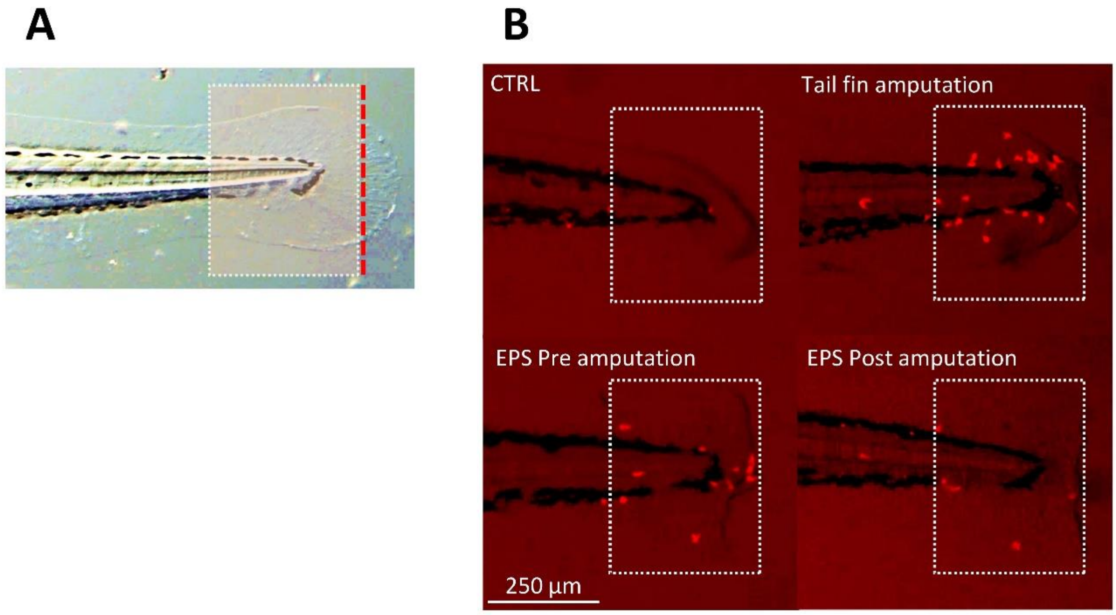

Figure S2. (**A**) Image of the tail of a 3 dpf zebrafish larvae, showing the position of the caudal tail amputation (red line) and the area designated for counting neutrophils number. (**B**) Representative fluorescence microscopy images of wound-induced migration of neutrophils, in the four experimental conditions.

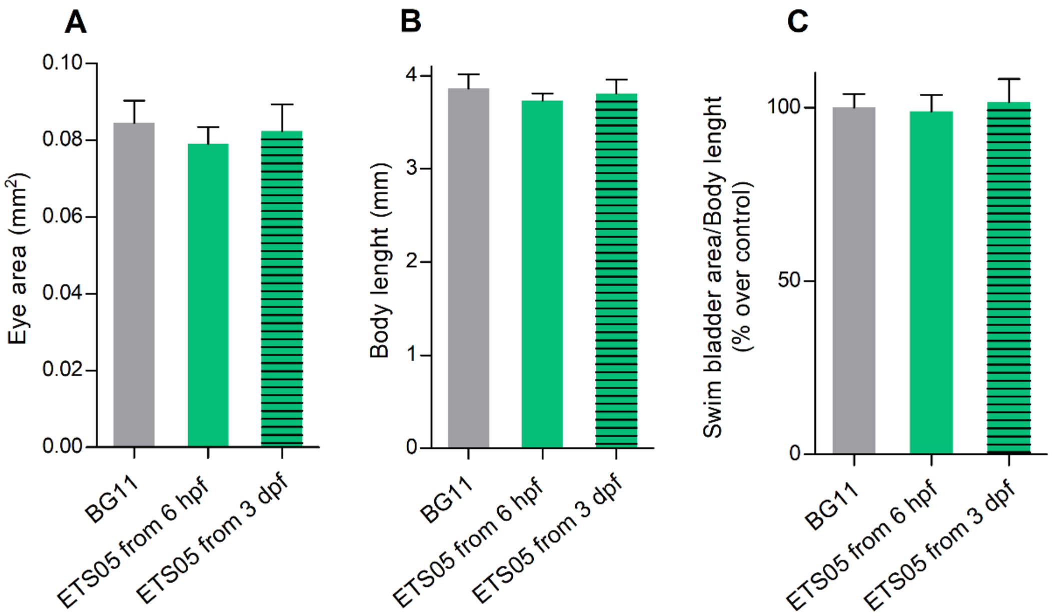

Figure S3. Morphological parameters analyzed on 5 dpf zebrafish larvae after co-culture with *Phormidium* at initial concentration of 0.3 (OD value) for either 5 days or 2 days. **A:** eye area; **B:** body length; **C:** swim bladder area. To avoid redundancy only results obtained with the higher *Phormidium* concentration used are shown. The data represent the mean±SD of three independent experiments each conducted with 12-15 larvae. Statistical analysis was performed using GraphPad Prism 7 (One-way ANOVA followed by Tukey's multiple comparison test).

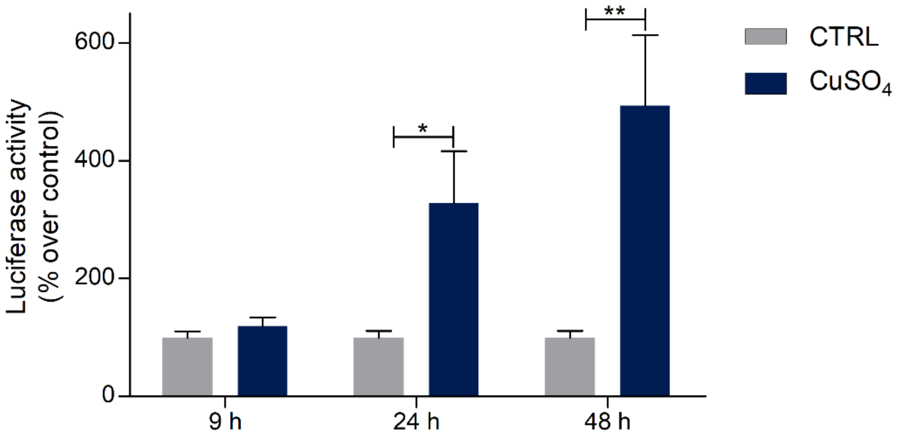

Figure S4. Copper treatment results in NF-κB pathway activation. Induction was analysed with the zebrafish transgenic line NFκB:GFP,Luc at three time points and was calculated by dividing luciferase values from treated larvae by values of untreated siblings. The data represent the mean±SEM of a single experiment conducted with 20 larvae. Statistical analysis was performed using GraphPad Prism 5 (Unpaired t test with Welch's correction). Statistical significance: * $p \leq 0.05$, ** $p \leq 0.01$.

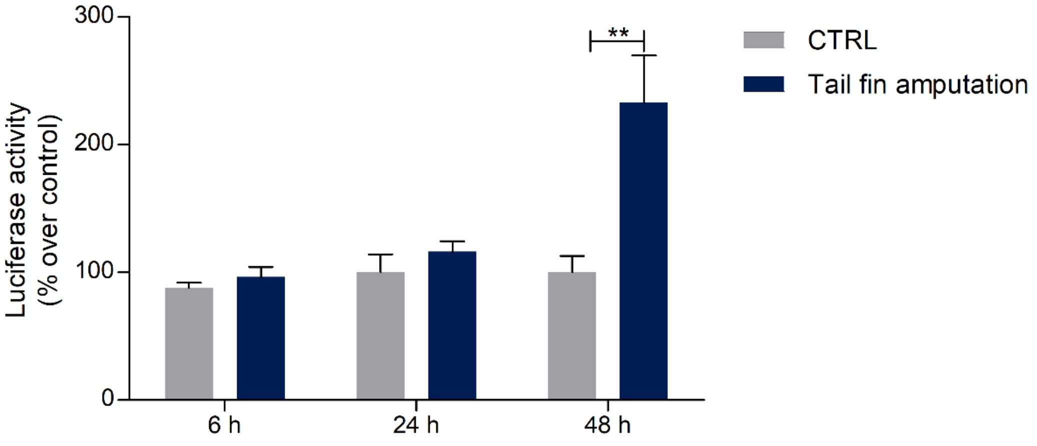

Figure S5. Caudal fin amputation results in NF-κB pathway activation. Induction of NF-κB activity was analyzed with the zebrafish transgenic line NFκB:GFP,Luc at three time points and was calculated by dividing luciferase values from treated larvae by values of untreated siblings. The data represent the mean±SEM of a single experiment conducted with 20 larvae. Statistical analysis was performed using GraphPad Prism 5 (Unpaired t test with Welch's correction). Statistical significance: ** $p \leq 0.01$.
